# Brain-Derived Neurotrophic Factor and Major Depressive Disorder: Evidence from Meta-Analyses

**DOI:** 10.3389/fpsyt.2017.00308

**Published:** 2018-01-17

**Authors:** Taro Kishi, Reiji Yoshimura, Toshikazu Ikuta, Nakao Iwata

**Affiliations:** ^1^Department of Psychiatry, School of Medicine, Fujita Health University, Toyoake, Japan; ^2^Department of Psychiatry, University of Occupational and Environmental Health, Kitakyushu, Japan; ^3^Department of Communication Sciences and Disorders, School of Applied Sciences, University of Mississippi, University, MS, United States

**Keywords:** brain-derived neurotrophic factor, pathophysiology, major depressive disorder, umbrella review, meta-analysis

## Abstract

Accumulating evidence suggests that brain-derived neurotrophic factor (BDNF) is associated with the pathophysiology of major depressive disorder (MDD). In this mini review, we explored the association between BDNF and MDD using meta-analytic evidence. Our findings indicated that the Val66Met polymorphism in the BDNF gene was not associated with MDD or hippocampal volume in patients with MDD. However, plasma/serum levels of BDNF were decreased in patients with acute MDD compared with healthy controls. Both antidepressant treatment and electroconvulsive therapy increased plasma and serum levels of BDNF in patients with MDD. Val66Met polymorphism in the BDNF gene was associated with an antidepressant response in patients with MDD. Taken together, we did not detect any plausible evidence regarding Val66Met polymorphism in the BDNF gene contributing to a risk of MDD. However, peripheral BDNF levels are decreased in patients with MDD, and the polymorphisms are associated with treatment response. In conclusion, BDNF is best understood to be a biomarker for the state of MDD and its treatment response rather than a risk factor for MDD.

## Introduction

Brain-derived neurotrophic factor (BDNF) has often been suggested to contribute to the pathophysiology of major depressive disorder (MDD). BDNF plays a major role in neuronal growth and survival, serves as a neurotransmitter modulator, and contributes to neuronal plasticity, all of which are related to MDD. BDNF stimulates and controls the growth of new neurons from neural stem cells (i.e., neurogenesis), and BDNF protein and mRNA have been detected in various regions of the brain, including the olfactory bulb, cortex, hippocampus, basal forebrain, mesencephalon, hypothalamus, brainstem, and spinal cord (1). Therefore, it has been suggested that abnormalities in BDNF in the brain are associated with the pathophysiology of MDD.

Findings of studies on BDNF and MDD vary in terms of genetic expression and peripheral BDNF levels. The findings of these studies are not fully consistent, and some studies have reported a significant association between BDNF and MDD, whereas others have not. The aim of this study was to elucidate the source of this inconsistency and identify the aspects of association between BDNF and MDD. An umbrella review to explore the association between BDNF (serum/plasma BDNF concentration and BDNF gene) and MDD (pathophysiology and antidepressant response) using meta-analytic evidence was conducted.

## Methods

To identify the latest and most relevant studies, we searched the Scopus, MEDLINE, Cochrane library, and PsycINFO databases, with restrictions of English articles published from 2012 to September12, 2017 (most recent 5 years). The search terms included “brain-derived neurotrophic factor (OR BDNF)” AND “major depressive disorder (OR MDD)” AND “meta-analysis.”

## Results

### Study Characteristics

Of the 63 studies initially identified in our literature search, 46 duplicate studies were excluded; 4 were excluded after a review of the title/abstract and 2 after a full-text review (both were review articles). No studies were retrieved through a search of review articles (1–11). Finally, eight studies were selected for this review article (2–8, 11). The results from these meta-analyses were summarized in Table S1 in Supplementary Material.

### BDNF Gene versus the Pathophysiology of MDD

A biologically functional human single-nucleotide polymorphism (SNP) was found in the 5′ pro-region of the BDNF gene located on human chromosome 11q14.1, with a substitution of valine (Val) with methionine (Met) at codon 66 (rs6265) (12). The Met allele of the precursor peptide was associated with impaired intracellular trafficking of pro-BDNF into dendrites and vesicles and reduction in activity-dependent secretion. Therefore, Val66Met in the BDNF gene is considered to play a major role in the regulation of extracellular BDNF levels (13). Accordingly, because it has been considered that Val66Met in the BDNF gene may be associated with the pathophysiology of MDD, many association studies involving Val66Met in the BDNF gene and MDD have been conducted (4, 12). Li et al. conducted a meta-analysis to examine the association between rs6265 (also called Val66Met) and the clinical risk of mood disorder, including MDD and bipolar disorder (4). They demonstrated that Val66Met in the BDNF gene was not associated with MDD in European (odds ratio = 1.00; 95% confidence interval (95% CI) = 0.93–1.09; *p* = 0.69; *I*^2^ = 48.4%; 24 case–control samples; 15,419 patients and 29,007 controls) and Asian (odds ratio = 0.97; 95% CI = 0.89–1.06; *p* = 0.535; *I*^2^ = 37.2%; 13 case–control samples and 1 family-based sample; 7,371 patients, 8,742 controls, and 105 trios) populations. Val66Met in the BDNF gene was indeed associated with bipolar disorder in the European population (odds ratio = 1.14; 95% CI = 1.04–1.23; *p* = 0.0029; *I*^2^ = 49.3%; 15 case–control samples and 4 family-based samples; 11,723 patients and 12,312 controls) but not in the Asian population (odds ratio = 0.97; 95% CI = 0.91–1.04; *p* = 0.443; *I*^2^ = 6.7%; 11 case–control samples; 3,324 patients and 3,314 controls). Tsang et al. conducted another meta-analysis and confirmed the association between Val66Met in the BDNF gene and the clinical risk of late-life depression (odds ratio = 1.33; 95% CI = 1.05–1.68; *p* = 0.02; *I*^2^ = 20%; *N* = 4, 577 patients and 425 control) (11). Thus, despite the lack of evidence for the association between the risk of MDD and Val66Met in the BDNF gene due to a limited number of studies, Val66Met in BDNF gene has been considered to be associated with late-life depression.

### BDNF Gene versus Hippocampal Volume in Patients with MDD

Hippocampal volume was consistently found to be reduced in MDD (14–16). Among healthy subjects, both the left and right hippocampal volumes were significantly larger in Val/Val homozygous carriers than in Met carriers [left: standardized mean difference (SMD) = 0.41, 95% CI = 0.20 to 0.62, *p* = 0.0001; right: SMD = 0.41, 95% CI = 0.20 to 0.61, *p* = 0.0001; *N* = 7, *n* = 399] (17). Harrisberger et al. performed a meta-analysis to examine the association between Val66Met in the BDNF gene and hippocampal volume in patients with psychiatric disorders, such as MDD (3). Val66Met in the BDNF gene was not associated with hippocampal volume in patients with MDD (Hedge’s *g* = 0.08; 95% CI = −0.05 to 0.22; *p* = 0.23; *I*^2^ = 0.00%; *N* = 8, *n* = 903) (3).

### Serum/Plasma Level of BDNF versus the Pathophysiology of MDD

Brain-derived neurotrophic factor can also be assessed in components other than those in the central nervous system. BDNF in plasma or serum is released from blood platelets (18); it is derived from the brain but has to cross the blood–brain barrier (19). In a meta-analysis, Polyakova et al. examined whether there was any significant difference in serum/plasma levels of BDNF between patients with MDD and healthy controls (5). BDNF levels were significantly decreased in patients with acute MDD (Cohen’s *d* = −0.80; 95% CI = −1.05 to −0.54; *p* < 0.0001; *I*^2^ = 91.2%; *N* = 38, *n* = 2,447). The studies were divided into serum- or plasma-based studies depending on the measurement of serum or plasma BDNF levels. BDNF level was significantly decreased in patients with acute MDD in serum-based studies (Cohen’s *d* = −0.81; 95% CI = −1.05 to −0.56; *p* < 0.0001; *I*^2^ = 91.5%; *N* = 32, *n* = 2,298), but not in plasma-based studies (Cohen’s *d* = −0.71; 95% CI = −1.55 to 0.13; *p* = 0.097; *I*^2^ = 91.1%; *N* = 6, *n* = 149); this may be due to the smaller sample size. In addition, serum/plasma BDNF levels were significantly decreased in patients with acute depressive episodes of bipolar disorder (Cohen’s *d* = −1.16; 95% CI = −1.79 to −0.54; *p* < 0.0001; *I*^2^ = 83.4%; *N* = 6, *n* = 117) and acute manic episodes of bipolar disorder (Cohen’s *d* = −0.77; 95% CI = −1.10 to −0.44; *p* < 0.0001; *I*^2^ = 50.0%; *N* = 8, *n* = 156). Peripheral BDNF levels were decreased in patients with MDD patients but were not specific to MDD or the state of depression.

### Serum/Plasma Level of BDNF versus Antidepressant Treatment in MDD

Zhou et al. conducted a meta-analysis to examine the association between serum/plasma concentrations of BDNF and treatment with antidepressants in patients with MDD (8). Treatment with antidepressants significantly increased serum/plasma BDNF levels (SMD = 0.62, 95% CI = 0.31 to 0.94, *p* < 0.0001, *I*^2^ = 85%; *N* = 20, *n* = 1,266). When stratified by antidepressant class, the selective serotonin reuptake inhibitor (SSRI) treatment subgroup exhibited increased serum/plasma BDNF levels (SMD = 0.46, 95% CI = 0.20 to 0.72, *p* = 0.0006, *I*^2^ = 71%; *N* = 17, *n* = 970), but the serotonin–noradrenaline reuptake inhibitor (SNRI) subgroup did not (SMD = 0.99, 95% CI = −0.05 to 2.04, *p* = 0.06, *I*^2^ = 94%; *N* = 5, *n* = 296). The lack of significant differences in the SNRI subgroup may be due to the smaller sample size. When stratified by the type of study (serum and plasma studies), BDNF was significantly increased in both serum (SMD = 0.68, 95% CI = 0.27 to 1.10, *p* = 0.001, *I*^2^ = 80%; *N* = 12, *n* = 540) and plasma studies (SMD = 0.92, 95% CI = 0.07 to 1.77, *p* = 0.03, *I*^2^ = 91%; *N* = 7, *n* = 308). Antidepressants, at least SSRIs, are suggested to elevate peripheral BDNF levels.

### Serum/Plasma Level of BDNF versus Electroconvulsive Therapy in MDD

Electroconvulsive therapy is often performed for pharmacological treatment-resistant MDD. Rocha et al. conducted a meta-analysis to examine the association between serum/plasma level of BDNF and electroconvulsive therapy in patients with MDD (6). Electroconvulsive therapy significantly increased serum/plasma BDNF levels (SMD = 0.56; 95% CI = 0.17 to 0.96; *p* = 0.006; *I*^2^ = 73%; *N* = 9, *n* = 414). However, sensitivity and meta-regression analyses did not detect any causes for this significant heterogeneity. Nevertheless, electroconvulsive therapy has been suggested to elevate peripheral BDNF levels.

### Serum/Plasma Level of BDNF versus Non-invasive Brain Stimulation Intervention in MDD

Non-invasive brain stimulation has been proposed as a new non-pharmacological technique to treat MDD. However, the effectiveness of this treatment has not been conclusively demonstrated (20). Brunoni et al. conducted a meta-analysis to examine the association between serum/plasma levels of BDNF and non-invasive brain stimulation intervention (repetitive transcranial magnetic stimulation and transcranial direct current stimulation) in patients with MDD (2). Brain stimulation interventions did not increase serum/plasma BDNF levels (SMD = 0.03; 95% CI = −0.21 to 0.27; *p* = 0.843; *I*^2^ = 0.00%; *N* = 8, *n* = 146). Neither repetitive transcranial magnetic stimulation nor transcranial direct current stimulation altered the serum/plasma BDNF levels (repetitive transcranial magnetic stimulation: SMD = 0.05, 95% CI = −0.30 to 0.39, *p* = 0.337, *I*^2^ = 11.1%, *N* = 4, *n* = 74; transcranial direct current stimulation: SMD = 0.00, 95% CI = −0.35 to 0.36, *p* = 1.00, *I*^2^ = 0.00%, *N* = 4, *n* = 72). Non-invasive brain stimulation did not reduce the serum/plasma BDNF levels, which may correspond to the absence of strong evidence for its efficacy.

### BDNF Gene versus Antidepressant Treatment Response in MDD

The Val66Met variation has been proposed as a risk factor for MDD and predictor for responses to antidepressant treatments. Yan et al. conducted a meta-analysis to examine the association between Val66Met in the BDNF gene and antidepressant treatment responses in patients with MDD (7). A greater number of Met carriers responded to antidepressants than patients with the Val/Val homozygous gene (odds ratio = 1.49; 95% CI = 1.05–2.12; *p* = 0.03; *I*^2^ = 57%; *N* = 14, *n* = 1,705). On treating Asians with SSRIs but not SNRIs (result not reported; *N* = 4, *n* = 394), more responders were detected among Met carriers than among Val/Val homozygous patients (odds ratio = 1.81; 95% CI = 1.10–2.97; *p* = 0.02; *I*^2^ = 66%; *N* = 9, *n* = 1,107). Although Val66Met may not be associated with the risk of MDD, it considerably predicts the responses to antidepressants.

## Discussion

This mini review explored evidence that indicated an association between BDNF levels and MDD. Val66Met in the BDNF gene did not show significant risk for MDD. However, Val66Met in the BDNF gene was associated with late-life depression. Epidemiological studies suggested that the heritability of MDD is approximately 40% (21). Although several genome-wide association studies (GWAS) of MDD have been conducted, these studies have not detected any markers associated with the clinical risk of MDD. Because the heritability of MDD is low, but its prevalence is high, the pathophysiology of MDD is considered to be highly heterogeneous. Therefore, GWAS could not detect any genetic markers associated with the clinical risk of MDD (i.e., the effect size of markers was extremely small) because of insufficient statistical power (22, 23). Because the meta-analysis of late-life depression was performed in only four studies (*n* = 1,002), and the effect size of Val66Met for late-life depression was relatively high, we considered that this positive result might be a type I error (11). Serum/plasma levels of BDNF were lower in patients with MDD than in healthy controls, and were increased after antidepressant treatment or electroconvulsive therapy. Therefore, although there was evidence suggesting that Val66Met is associated with late-life depression, BDNF can be considered as a marker for the state of MDD rather than a trait marker for MDD. However, it should be noted that Val66Met in the BDNF gene plausibly predicts the response to antidepressants in MDD. Moreover, further study with larger statistical power than that in previous studies is needed to examine whether Val66Met in BDNF gene is associated with late-life depression.

Surprisingly, although Val66Met in the BDNF gene was associated with antidepressant response in MDD, and serum/plasma levels of BDNF in patients with MDD increased after antidepressant treatment or electroconvulsive therapy, there was no significant difference in the frequency of polymorphisms in the BDNF gene between patients with MDD and healthy controls. The cause of heterogeneity might be the methodological quality of the studies, including age and gender matching and assay type (serum or plasma). Several researchers reported an absence of association between the BDNF gene and MDD, stating that the pathophysiology as well as diagnosis of MDD is extensively heterogeneous and, hence, it is very difficult to detect a susceptibility gene for MDD (4, 23).

It should be noted that Val66Met in the BDNF gene has been associated with episodic memory performance (13, 24) and anxiety/neuroticism (25, 26). The Val66Met polymorphism may not predict the risk for MDD; however, it has been shown to influence cognitive and psychological states associated with MDD. These commonly observed phenotypes in patients with MDD are associated with Val66Met in the BDNF gene.

Terracciano et al. reported that GWAS demonstrated an association between serum BDNF levels and rs11030102 in the BDNF gene, but not Val66Met (27). Therefore, additional meta-analytic studies are needed to examine whether other SNPs in BDNF are associated with MDD.

Recently, ketamine has been proposed as an attractive antidepressant (28). Ketamine is an *N*-methyl-d-aspartate receptor antagonist which upregulates the α-amino-3-hydroxy-5-methyl-4-isoxazolepropionic acid receptor and activates downstream neuroplasticity signaling pathways. It exhibits very acute therapeutic effects, including the acute removal of suicidal intents. Yan et al. reported that Val66Met in the BDNF gene was associated with antidepressant responses in patients with MDD (7). Met carriers had improved Hamilton Depression Rating Scale scores (24% reduction from baseline to endpoint) compared with Val carriers (41%) (29). The association between variations in the BDNF gene and ketamine remains unclear. Further studies will be required to attest the association between the BDNF gene and ketamine response in patients with MDD.

In conclusion, we could not find evidence which suggested that the Val66Met variation in the BDNF gene is a risk factor for MDD; however, the Val66Met variation in the BDNF gene may predict the responses to antidepressant. Peripheral BDNF levels have been confirmed to be markers for the state of MDD. It is implied that BDNF is not as relevant to the risk for MDD as to the state of MDD.

## Author Contributions

TK and RY had complete access to all the data used in the study. The study concept and design were performed by TK and RY. The manuscript was written by all the authors. NI supervised the review.

## Conflict of Interest Statement

TK, RY, TI, and NI declare that they have no direct conflicts of interest relevant to this study. No grant support or other sources of funding were used to conduct this study or prepare this manuscript. TK has received speaker’s honoraria from Daiichi Sankyo, Dainippon Sumitomo, Eisai, Janssen, Otsuka, Meiji, MSD, and Tanabe-Mitsubishi (Yoshitomi), and has received research grants from Health Labor Sciences and Fujita Health University School of Medicine. RY has received speaker’s honoraria from Eli Lilly, Janssen, Dainippon Sumitomo, Otsuka, Meiji, Pfizer, Shionogi, and Yoshitomi. TI has received speaker’s honoraria from Eli Lilly, Daiichi Sankyo, and Dainippon Sumitomo. NI has received speaker’s honoraria from Astellas, Dainippon Sumitomo, Eli Lilly, GlaxoSmithKline, Janssen, Yoshitomi, Otsuka, Meiji, Shionogi, Novartis, and Pfizer, and has received research grants from GlaxoSmithKline, Meiji, and Otsuka.
