# Supplementary material for: Brain-Derived Neurotrophic Factor and Major Depressive Disorder: Evidence from Meta-Analyses
Source: Front Psychiatry. 2018 Jan 17;8:308. doi: 10.3389/fpsyt.2017.00308 (PMC5776079; doi:10.3389/fpsyt.2017.00308)
Supplement: Supplementary file 1 [file Table_1.docx]

**Table S1. Summary of Evidence from Meta-analyses**

| Val66Met vs. MDD (European population) | OR = 1.00, 95% CI = 0.93–1.09, p = 0.69, I^2^ = 48.4%; 24 case-control samples; 15,419 patients and 29,007 controls |
| --- | --- |
| Val66Met vs. MDD (Asian population) | OR = 0.97, 95% CI = 0.89–1.06, p = 0.535, I^2^ = 37.2%; 13 case-control samples and 1 family-based samples; 7,371 patients, 8,742 controls and 105 trios |
| Val66Met vs. late-life depression | OR = 1.33, 95% CI = 1.05–1.68, p = 0.02, I^2^ = 20%; N = 4, 577 patients and 425 controls |
| Val66Met vs. hippocampal volumes in MDD | Hedge’s g = 0.08, 95% CI = −0.05 to 0.22, p = 0.23, I^2^ = 0.00%; N = 8, n = 903 |
| Serum/plasma BDNF level vs. acute MDD | Cohen's d = −0.80, 95% CI = −1.05 to −0.54, p < 0.0001, I^2^ = 91.2%; N = 38, n = 2,447 |
| Serum/plasma BDNF level vs. antidepressant treatment in MDD | SMD = 0.62, 95% CI = 0.31to 0.94, p < 0.0001, I^2^ = 85%; N = 20, n = 1,266 |
| Serum/plasma BDNF level vs. electroconvulsive therapy in MDD | SMD = 0.56, 95% CI = 0.17 to 0.96, p = 0.006, I^2^ = 73%; N = 9, n = 414 |
| Serum/plasma BDNF level vs. noninvasive brain stimulation intervention in MDD | SMD = 0.03, 95% CI = –0.21 to 0.27, p = 0.843, I^2^ = 0.00%; N = 8, n = 146 |
| Val66Met vs. antidepressant treatment response in MDD | OR = 1.49, 95% CI = 1.05–2.12, p = 0.03, I^2^ = 57%; N = 14, n = 1,705 |

95% CI: 95% confidence interval, BDNF: brain-derived neurotrophic factor, MDD: major depressive disorder, N: number of studies, n: number of patients and controls, OR: odds ratio, SMD: standardized mean difference
